# Fluorescence-Guided Bladder and Ureter Visualization in Benign Robotic Gynecologic Surgery: A Series of Five Cases

**DOI:** 10.7759/cureus.98847

**Published:** 2025-12-09

**Authors:** Urmila Soman, Megi Cela

**Affiliations:** 1 Robotic and Minimally Invasive Gynecology, Aster Medcity, Kochi, IND

**Keywords:** benign, bladder, icg, robotic surgery, ureter

## Abstract

Indocyanine green (ICG) near-infrared fluorescence imaging is well established in gynecologic oncology, but its role in benign minimally invasive surgery is still limited and not standardized. Increasing cesarean section and endometriosis rates elevate the risk of urinary tract injury, highlighting the need for improved intraoperative visualization. This case series evaluates the practical use and reliability of ICG fluorescence for bladder and ureteral identification during benign robotic gynecologic procedures.

Two patients undergoing robotic hysterectomy using the Da Vinci Xi Firefly system (Intuitive Surgical, Sunnyvale, California, United States) for benign indications had a history of prior cesarean deliveries. Intravesical ICG was administered to delineate the bladder. However, fluorescence was patchy and unreliable, with only partial bladder wall visualization despite adequate technique.

In contrast, two patients with deep infiltrating endometriosis involving the ureter underwent cystoscopic retrograde instillation of ICG, which provided consistent and clear fluorescence-guided ureteral mapping, facilitating safe dissection and preservation. A fifth case involved a patient with symptomatic adenomyosis and an endometriotic ovarian cyst, in whom preoperative imaging also revealed an ectopic left kidney, requiring careful retroperitoneal assessment and ureteral identification during robotic hysterectomy.

All surgeries were completed robotically without complications. Ureteral visualization with ICG was reproducible and effective, while bladder demarcation using intravesical ICG showed variable fluorescence, likely due to fibrosis and impaired diffusion in scarred tissue.

Fluorescence-guided robotic surgery using ICG is a valuable adjunct for ureteral identification in benign gynecology, particularly in advanced endometriosis. However, its application for bladder demarcation after multiple cesarean sections appears less reliable, showing the need for protocol improvement and further evaluation before routine use.

## Introduction

Near-infrared fluorescence with indocyanine green (ICG) is well established in gynecologic oncology, especially for sentinel lymph node mapping, but its use in benign gynecology is still evolving [1]. As minimally invasive approaches become routine, surgeons increasingly explore ICG to help identify structures such as the bladder and ureters, assess perfusion, and guide dissection [2]. Retrograde ureteral instillation introduces ICG directly into the ureteral lumen, where the dye remains intraluminal due to the impermeability of the urothelium. Under near-infrared imaging, this produces a clear fluorescent outline of the ureter for accurate anatomical localization. Intravesical instillation similarly confines ICG to the bladder lumen, coating the mucosal surface and generating uniform fluorescence that delineates bladder contours. This becomes particularly relevant in patients with distorted pelvic anatomy, most commonly due to previous cesarean deliveries or deep infiltrating endometriosis, where the risk of urinary tract injury is noticeably higher. Ureteral injury is a recognized complication during complex gynecologic procedures, particularly in patients with deep endometriosis or extensive adhesions. Robotic-assisted ureterolysis using ICG has been reported as a feasible technique to improve intraoperative ureter visualization [3]. Rare anatomic variants, such as ectopic kidneys with atypical ureteral pathways, further increase the challenge of pelvic dissection and highlight the importance of reliable intraoperative visualization strategies. Subsequent studies have applied ICG-guided ureterolysis in complex laparoscopic or robotic gynecologic surgeries, and systematic intraureteral ICG injection has been proposed as a reliable method to reduce the risk of injury [4,5].

Despite growing interest, protocols for ICG use in benign gynecologic surgery remain inconsistent. Most of the available literature consists of small case reports or heterogeneous case series, which describe institution-specific techniques rather than systematically evaluated protocols. In this case series, we present two patients who received intravesical ICG for bladder demarcation during robotic hysterectomy and three patients who underwent retrograde ureteric ICG instillation for endometriosis surgery using the Da Vinci Xi Firefly system (Intuitive Surgical, Sunnyvale, California, United States), an imaging mode on the Da Vinci robotic system that detects ICG within tissues or lumens. The goal is to compare the reliability and practicality of each technique in a benign gynecology setting.

## Case presentation

Cases 1 and 2: bladder demarcation using intravesical ICG

Two patients undergoing robotic hysterectomy for benign indications had a history of prior cesarean sections. To facilitate dissection of the vesicouterine plane, intravesical ICG was administered (25 mg diluted in 200 mL of normal saline and sterile water) through a Foley catheter, which was temporarily clamped for five and 10 minutes, respectively.

Case 1

**Figure 1:**
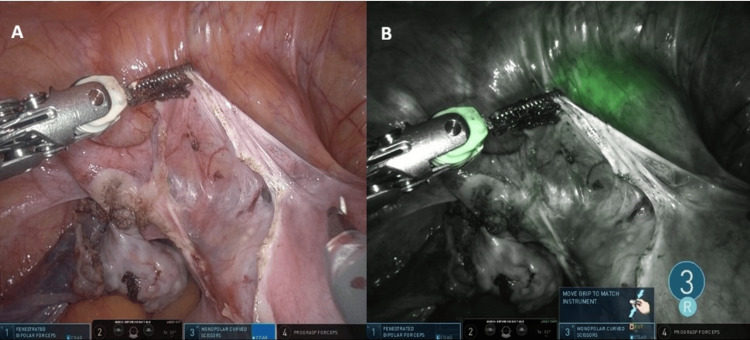
(A) Initial dissection of the vesicouterine space before Firefly activation, showing opened vesicouterine peritoneum and anterior fibrotic bands from prior cesarean delivery. (B) Firefly near-infrared imaging demonstrating patchy and incomplete bladder wall fluorescence after intravesical indocyanine green instillation, with non-fluorescent scarred regions superiorly

The patient was a 48-year-old woman with a history of two cesarean deliveries who underwent robotic hysterectomy with bilateral salpingectomy for multiple uterine fibroids associated with abnormal uterine bleeding. The appearance of the vesicouterine dissection and the subsequent bladder fluorescence pattern are shown in Figure 1.

Case 2

**Figure 2:**
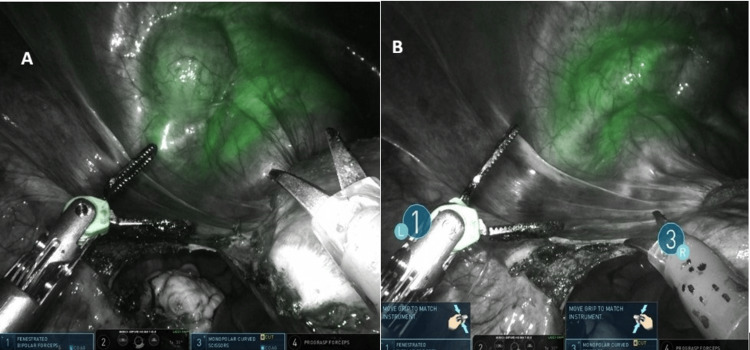
Firefly near-infrared imaging showing patchy and incomplete bladder wall fluorescence after intravesical indocyanine green instillation. Non-fluorescent scarred regions are visible superiorly in both images A and B

The patient was a 41-year-old woman with a history of one cesarean delivery who underwent robotic hysterectomy with bilateral salpingectomy for grade 3 uterine prolapse. The intraoperative view before fluorescence activation and the bladder fluorescence obtained after ICG instillation are presented in Figure 2. 

Both cases demonstrated inconsistent and patchy fluorescence, with partial visualization limited to non-scarred areas of the bladder wall. Fluorescence was poor in regions with dense fibrosis. Despite the suboptimal imaging, both surgeries were completed robotically without injury to the bladder or ureters.

Cases 3 and 4: ureter visualization using retrograde ICG

Two patients with advanced endometriosis underwent robotic surgery. Due to significant adhesions, ureteric identification was expected to be challenging. Cystoscopic retrograde instillation of ICG (12.5 mg diluted in 5 mL normal saline) was performed into each ureter.

Case 3

**Figure 3:**
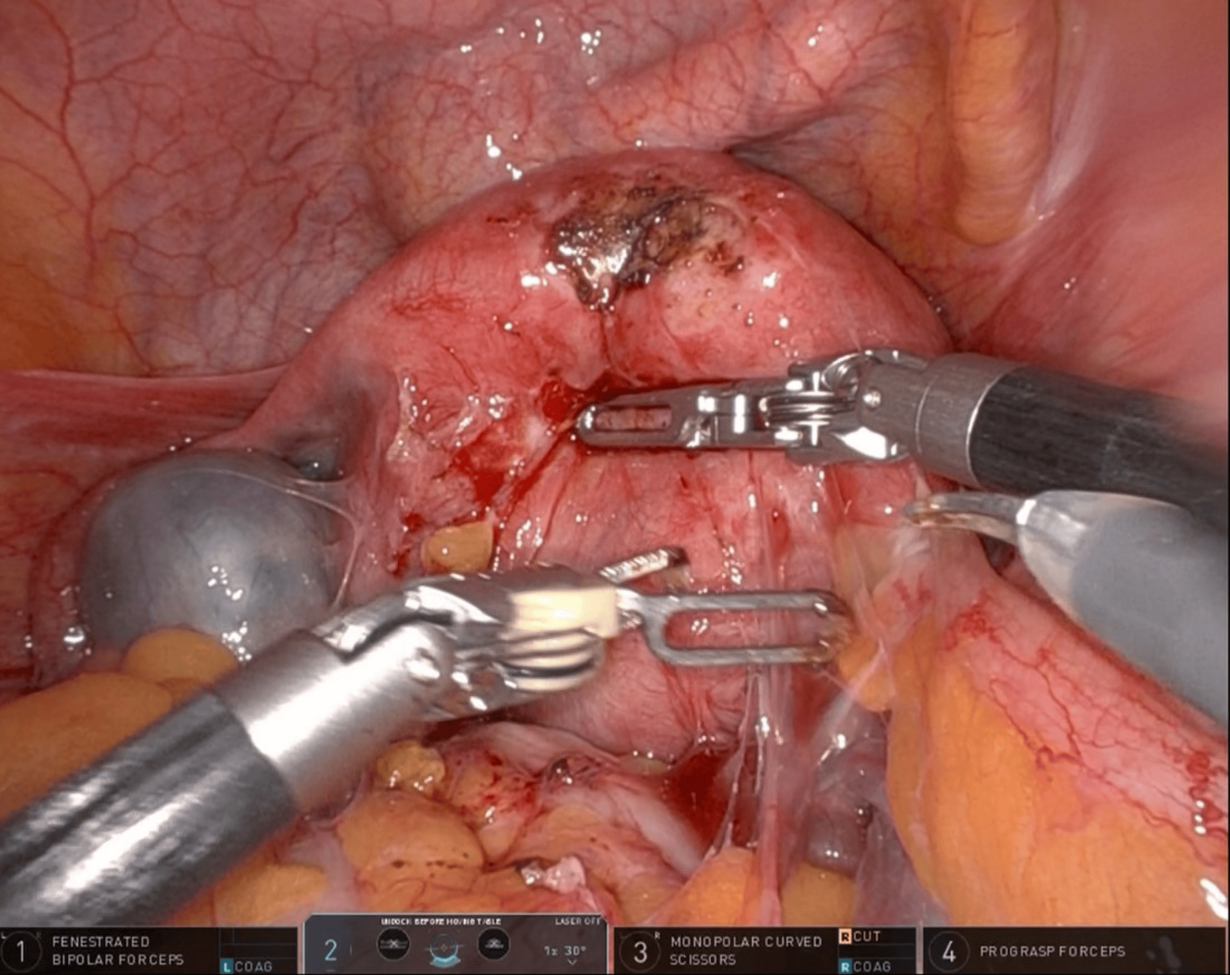
Robotic exposure of DIE case with posterior compartment obliterated and distorted anatomy planes

**Figure 4:**
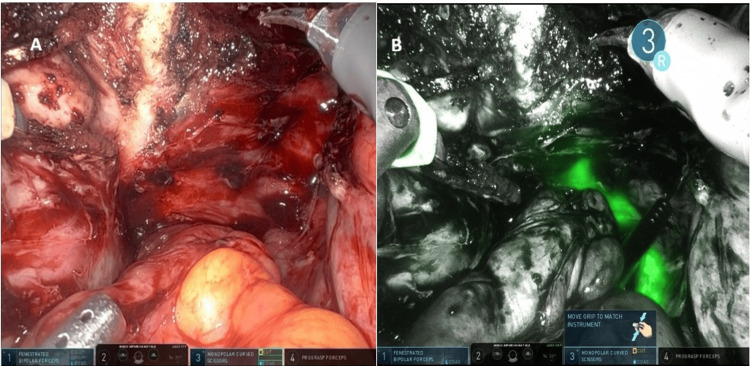
(A) Right pelvic sidewall following the excision of deep posterior endometriosis, demonstrating restored anatomical landmarks. (B) Firefly near-infrared imaging confirming ureteral integrity following dissection with continuous indocyanine green fluorescence outlining the ureter. No areas of interruption or leakage are observed, confirming preserved perfusion and structural integrity

**Figure 5:**
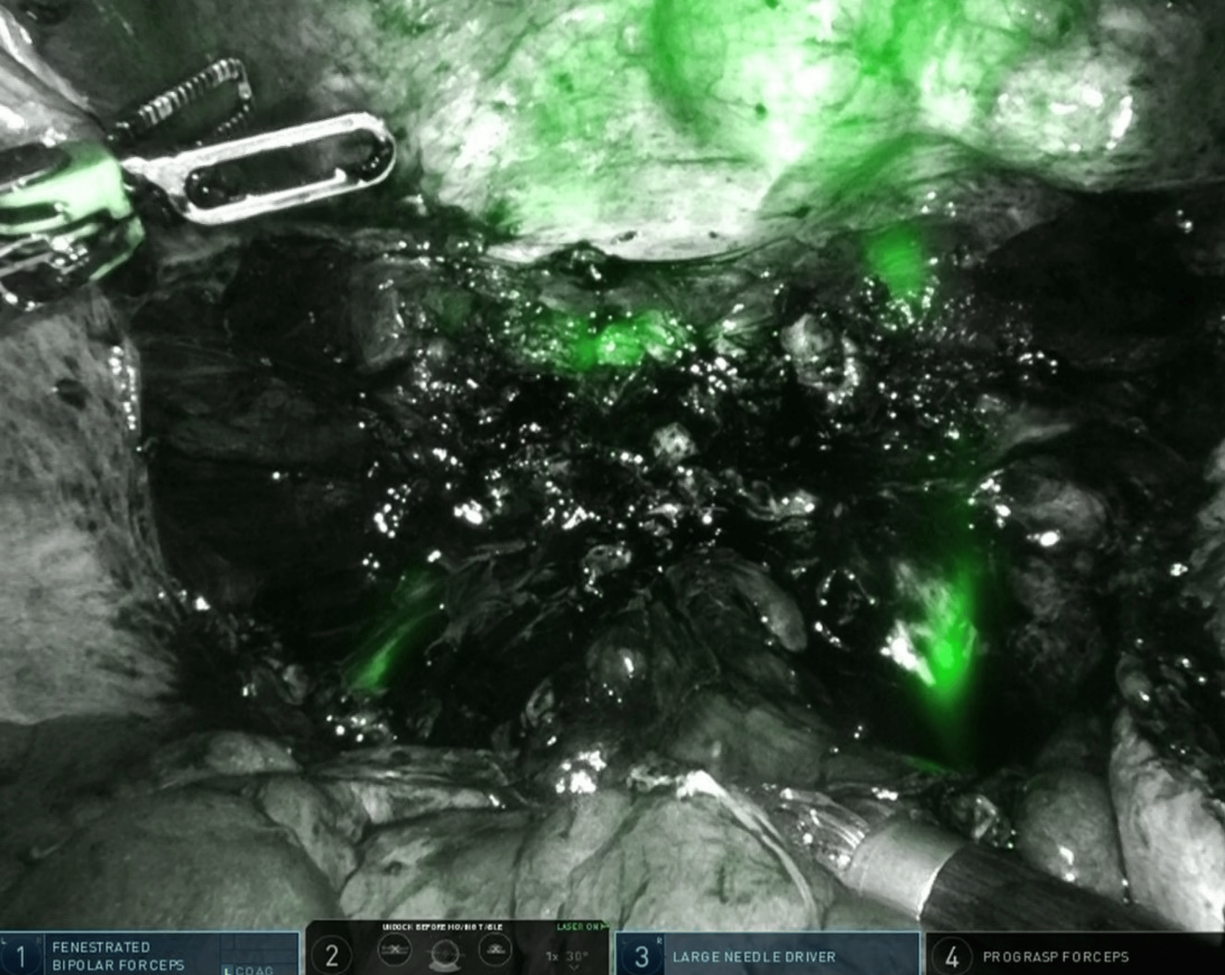
Firefly near-infrared fluorescence imaging of the ureter following hysterectomy and excision of endometriosis. At the end of the surgery, Firefly mode confirms clear visualization of the ureteral course within the retroperitoneum, demonstrating intact anatomy and preserved vascularity with no evidence of injury. This final fluorescence assessment highlights the role of indocyanine green guidance in ensuring ureteral safety during complex endometriosis surgery

The patient was a 43-year-old woman with deep infiltrating endometriosis who underwent a robotic hysterectomy with bilateral salpingo-oophorectomy. Preoperative MRI revealed extensive deep pelvic endometrial implants and demonstrated the right ureter in close proximity to the posterior uterine wall. Intraoperative findings and ureteral fluorescence obtained during dissection are shown in Figures 3-5.

Case 4

**Figure 6:**
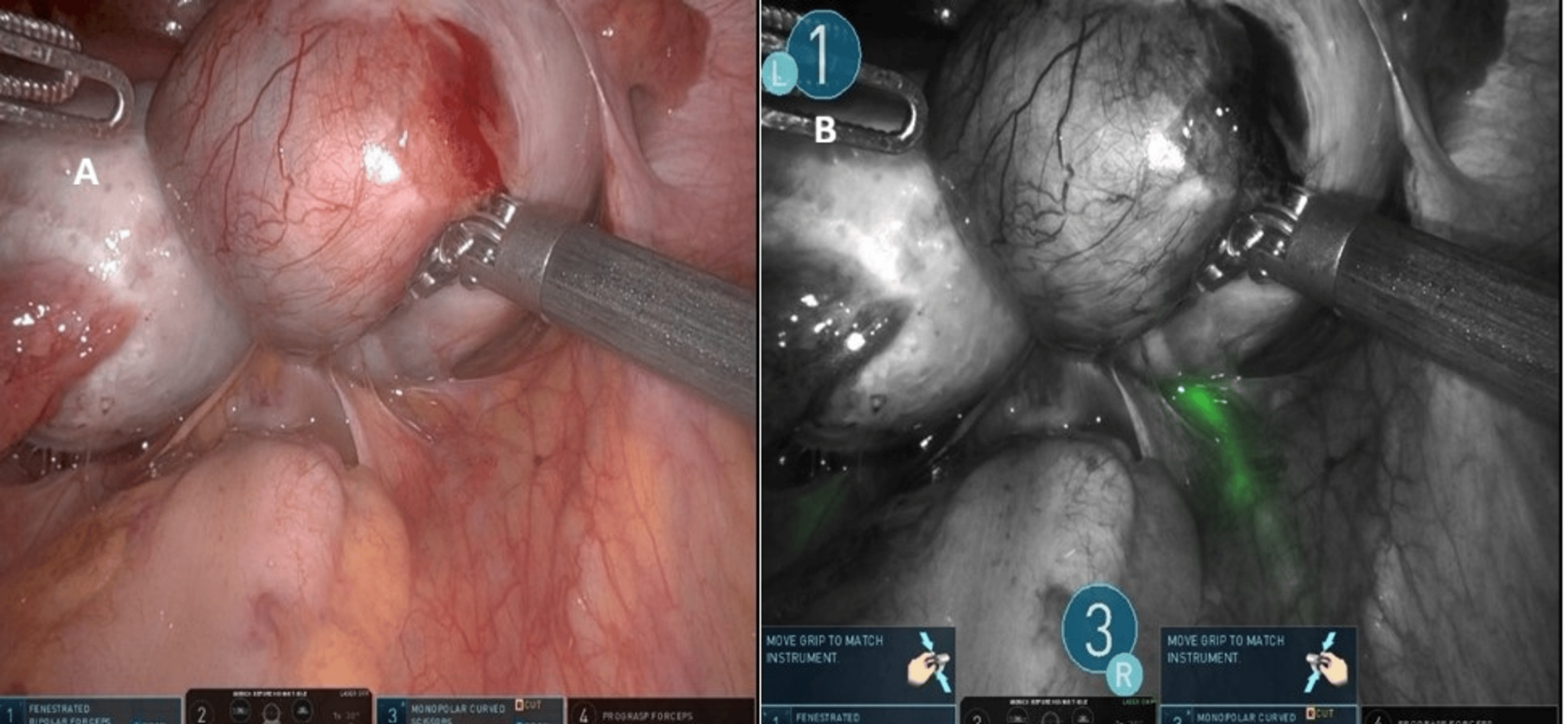
(A) Standard white-light view at the beginning of surgery, showing distorted adnexal anatomy with kissing ovaries. (B) Firefly near-infrared imaging after cystoscopic retrograde indocyanine green instillation, highlighting the ureteral pathway for early intraoperative identification during mobilization of the pelvic sidewall

**Figure 7:**
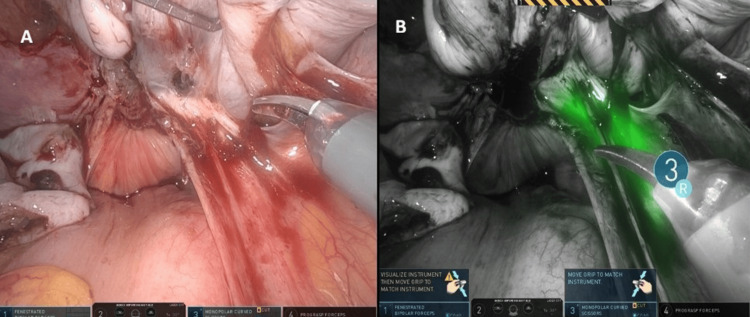
(A) White-light visualization of the right pelvic sidewall and obliterated posterior compartment during endometriosis excision. (B) Corresponding Firefly near-infrared view outlining the right ureter beneath the peritoneum, aiding safe posterior dissection. This fluorescence-supported identification reinforces surgical safety by ensuring continuous visualization of the ureter during management of deep endometriosis

**Figure 8:**
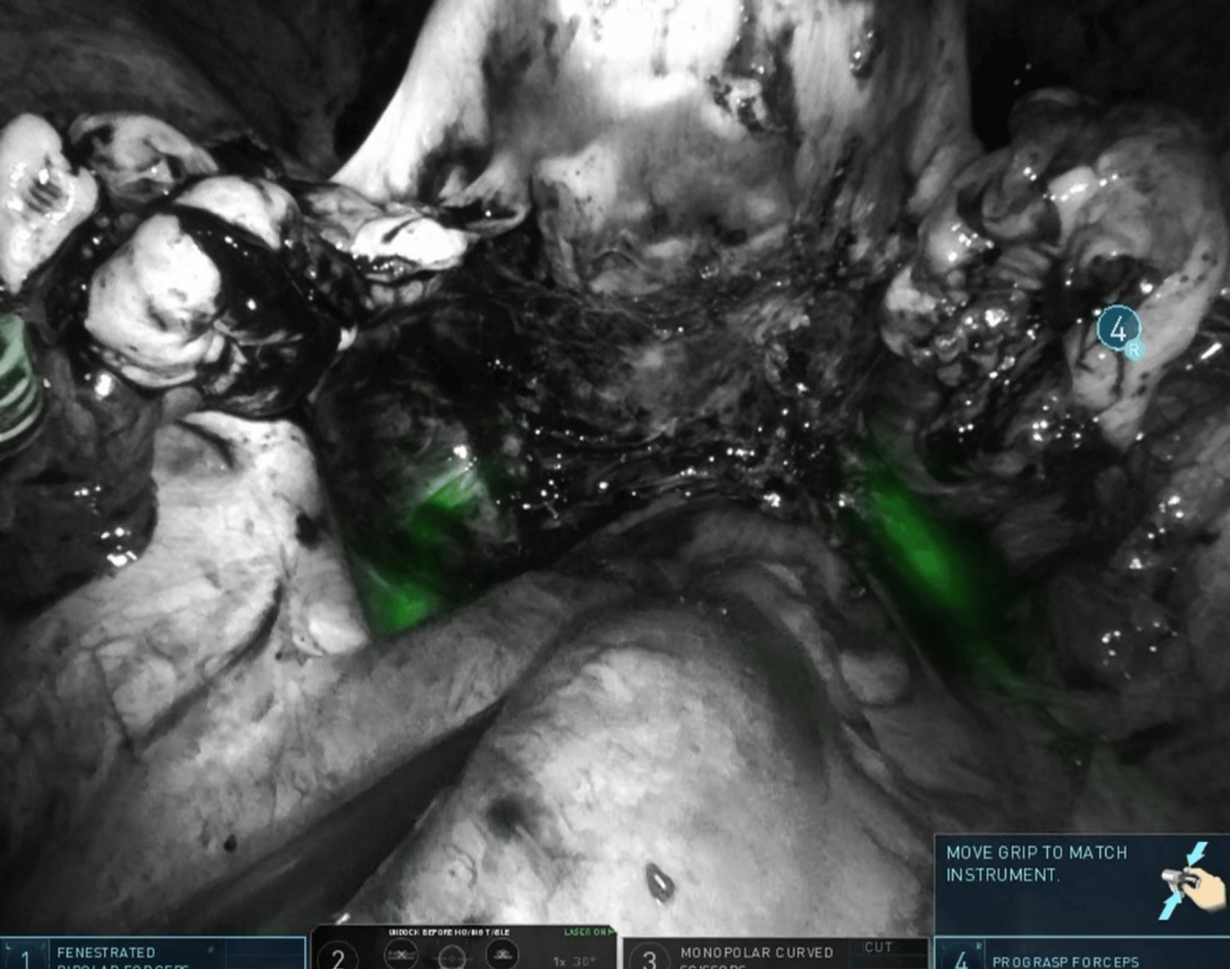
Final inspection at the completion of endometriotic disease excision and pelvic sidewall dissection. The ureters are clearly visualized confirming their integrity and anatomical path. Fluorescence guidance provides real-time confirmation of ureteral preservation before closure

A 29-year-old patient with stage 3 endometriosis underwent robotic endometriosis excision. Preoperative MRI demonstrated a multiloculated cystic lesion in the right adnexa consistent with a tubo-ovarian endometrioma, a unilocular left ovarian endometrioma, and kissing ovaries. The intraoperative white-light and Firefly near-infrared fluorescence views used for ureteral identification throughout the procedure are presented in Figures 6-8.

Both procedures were completed without complications.

Case 5: ureter visualization with ICG in a patient with an ectopic kidney

**Figure 9:**
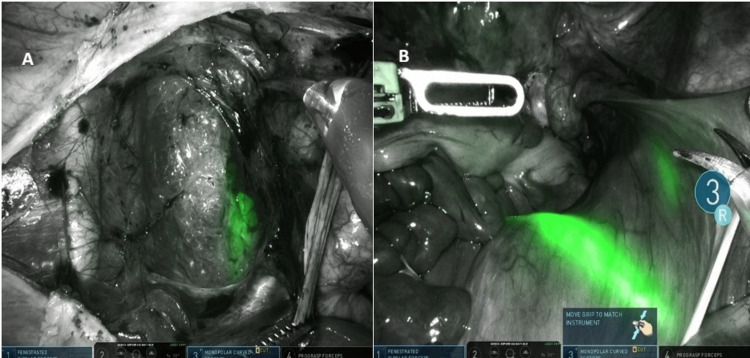
(A) Near-infrared fluorescence at the beginning of surgery showing clear visualization of the distal left ureter. (B) Fluorescence demonstrating both ureters entering the pelvis on the patient's right side, consistent with the ectopic renal anatomy

**Figure 10:**
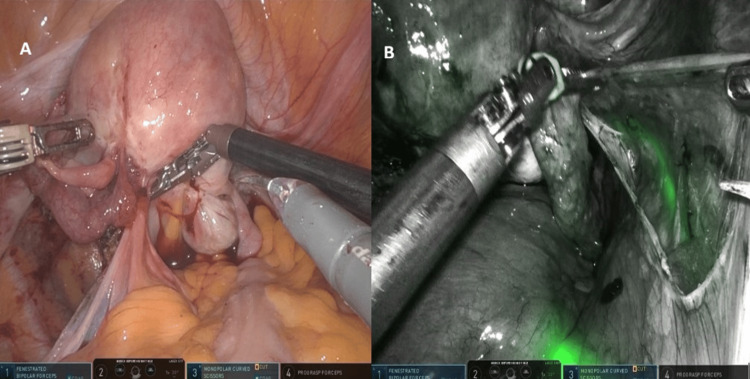
(A) Dense adhesions distorting posterior compartment anatomy, including bowel adhesions, adnexal adhesions, and a right ovarian endometriotic cyst. (B) Indocyanine green fluorescence aiding the identification of the ureteral course and facilitating entry into the correct dissection planes despite abnormal anatomy

**Figure 11:**
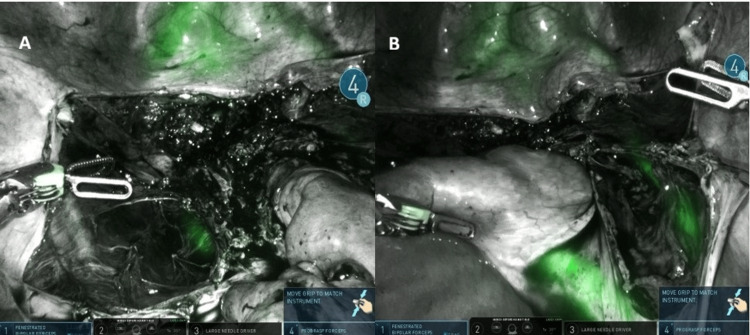
(A) End-of-procedure view confirming the left ureter coursing toward and entering the bladder. (B) Final fluorescence assessment showing both ureters intact and in their original anatomic positions without injury

A 50-year-old patient was scheduled for robotic hysterectomy with bilateral salpingo-oophorectomy due to symptomatic adenomyosis and chronic pelvic pain. Preoperative MRI demonstrated posterior wall adenomyosis, multiple intramural fibroids, a right ovarian endometriotic cyst, and a left hydrosalpinx. An additional significant finding was the presence of an ectopic left kidney, located inferior and medial to the right kidney. The ectopic kidney showed no hydronephrosis. Ureteral anatomy was presumed to be atypical due to the ectopic renal position, raising concern for potential ureteral vulnerability during pelvic dissection. Intraoperative ICG near-infrared fluorescence imaging confirmed the altered course of the ureters at the beginning of surgery (Figure 9A-9B), assisted in entering distorted posterior planes (Figure 10A-10B), and verified bilateral ureteral integrity at the end of the procedure (Figure 11A-11B).

## Discussion

This case series highlights the difference in reliability between intravesical and retrograde ICG application in benign gynecologic robotic surgery. Intravesical ICG showed limited and inconsistent uptake in both patients with prior cesarean deliveries. The most likely explanation is that cesarean-related fibrosis restricts ICG diffusion through the bladder mucosa and reduces near-infrared penetration, resulting in uneven fluorescence. Other authors have noted similar challenges, particularly in scarred anterior compartments where visualization is clinically most important [6].

In contrast, retrograde ICG instillation provided uniform and strong fluorescence in both patients undergoing endometriosis surgery. Because the dye directly contacts the ureteral mucosa, uptake is more predictable, and the linear anatomy of the ureter produces a continuous signal. This significantly improves safety during ureterolysis, especially when fibrosis has distorted normal landmarks. The findings align with existing literature demonstrating the effectiveness of ureteric ICG in complex benign pelvic surgery [1,7].

The fifth case illustrated how congenital renal anomalies can alter ureteral pathways and increase the complexity of pelvic surgery. In this patient, the ectopic left kidney resulted in both ureters originating on the right side of the pelvis, raising concern for potential misidentification during dissection. Intraoperative ICG fluorescence allowed the clear visualization of the atypical ureteral course at the beginning of the procedure and confirmed bilateral ureteral continuity into the bladder at the end of the surgery. This case highlights the utility of fluorescence imaging not only in diseased or fibrotic retroperitoneal spaces but also in patients with unusual anatomic configurations, consistent with previous reports describing the use of ICG to localize ureters in cases of crossed renal ectopia [8].

For trainees, fluorescence offers a helpful visual guide during robotic surgery, where tactile feedback is limited. However, the current lack of standardized dosing, dilution, and technique, especially for intravesical use, remains a barrier to broader adoption.

## Conclusions

ICG fluorescence is a valuable adjunct in benign robotic gynecologic surgery. Retrograde ureteric ICG provides dependable visualization and is particularly useful in endometriosis cases requiring ureterolysis. The presence of unusual anatomic variants, such as ectopic renal positioning, further reinforces the need for dependable ureteral visualization tools to ensure safe dissection during complex gynecologic surgery. On the other hand, intravesical ICG for bladder demarcation shows inconsistent results in patients with previous cesareans, limiting its usefulness. Further evaluation in larger prospective studies is needed to clarify optimal protocols and define its role in benign gynecologic surgery.
